# Overview of Calcium Phosphates used in Biomimetic Oral Care

**DOI:** 10.2174/1874210601812010406

**Published:** 2018-05-31

**Authors:** Frederic Meyer, Bennett T. Amaechi, Helge-Otto Fabritius, Joachim Enax

**Affiliations:** 1 Dr. Kurt Wolff GmbH & Co. KG, Research Department, Johanneswerkstr. 34-36, 33611 Bielefeld, Germany; 2 Department of Comprehensive Dentistry, University of Texas Health Science Center, 703 Floyd Curl Drive, San Antonio, Texas 78229-3900, USA; 3 Max-Planck-Institut für Eisenforschung GmbH, Microstructure Physics and Alloy Design, Max-Planck-Straße 1, 40237 Duesseldorf, Germany

**Keywords:** Enamel, Remineralization, Caries, Erosion, Calcium phosphates, Hydroxyapatite, Amorphous calcium phosphate

## Abstract

**Background:**

The use of biomimetic agents is an emerging field in modern oral care. Promising biomimetic substances for such applications are calcium phosphates, because their chemical composition is very similar to that of the mineral phase in human teeth, especially of natural enamel. Examples for their application include the remineralization of early caries lesions and repair of small enamel defects.

**Objective:**

This review provides an interdisciplinary view on calcium phosphates and their applications in biomimetic oral care. The aim of this work is to give an overview of *in vivo* and *in situ* studies comparing several calcium phosphates in preventive dentistry that can be used as a knowledge base for the development of innovative alternative oral care concepts.

**Methods:**

Books, reviews, and original research papers with a focus on *in vivo* and *in situ* studies were included. The databases PubMed^®^ and SciFinder^®^ were used for literature search. Calcium phosphates that are frequently utilized in oral care products are covered in this review and were used as search terms alone and together with the following key words: *in vivo*, *in situ*, caries, clinical study, and remineralization. From 13,470 studies found, 35 studies complied with the inclusion criteria and were used for this review.

**Results:**

Published *in vivo* and *in situ* studies demonstrate calcium phosphates’ potential in enamel remineralization. However, more studies are needed to further substantiate existing results and to extend and refine the application of calcium phosphates in modern oral care.

**Conclusion:**

Calcium phosphates represent an innovative biomimetic approach for daily oral care because of their high similarity to natural enamel that will broaden the range of future treatments in preventive dentistry.

## 1 BACKGROUND

The external layer of human teeth, the enamel, consists of micrometer-sized carbonated calcium-deficient hydroxyapatite (bioapatite) crystallites which are tightly packed and hierarchically organized in crystallite bundles (enamel prisms). Compared to other hard tissues in the human body (*i.e*. dentin and bone), enamel is harder and has a higher degree of mineralization as well as larger crystallites. According to thermogravimetric analysis it contains about 97% Hydroxyapatite (HAP) and only about 1.5% proteins (mainly collagen), and 1.5% water [1, 2]. Therefore, enamel represents the hardest tissue in the human body. Enamel has remarkable mechanical properties such as elaborating fracture toughness and high hardness due to its biocomposite character and a unique structural organization [1-4]. Like many other hard tissues occurring in living organisms [3, 5], teeth are formed in a complex biomineralization process by specialized cells under genetic control. While the general process in many natural systems (including *e.g*. human bones and teeth, Sponges, Molluscs, and Crustacea) is quite well understood, detailed knowledge is often still limited to date, especially in the realm of proteins involved. Proteins are essential for controlling biomineralization processes and each of these systems requires its own set of different proteins. To recapitulate the enamel formation process, organization of enamel crystallites takes place before a tooth erupts [6, 7]. Besides other cells, mainly ameloblasts produce the known non-collagenous proteins that are involved in amelogenesis, such as amelogenin, enamelin, ameloblastin, amelotin, and certain proteinases that are encoded by *AMEL*, *ENAM*, and *AMBN*. All of these genes belong to the Secretory Calcium binding Phosphoprotein gene family (SCPP) arising from the same single ancestor gene *SPARCL1* [6, 8]. Ameloblasts incorporate calcium and phosphate. In metabolic pathways where the proteins mentioned above are involved, intermediate stages and finally HAP-crystals originate [7]. Every ameloblast generates a Tomes-appendix growing in the dentine-direction. Around these appendices, apatite-crystals organize into prisms that constitute the enamel. As soon as a certain thickness of enamel is reached, the Tomes-appendices are formed back. The ameloblasts now convert to the terminal epithelium representing the *cuticula dentis*. After eruption of teeth, this *cuticula dentis* gets eroded by shear forces [7]. Consequently, the enamel layer of erupted teeth cannot regenerate and is supposed to last life-long. Approaches to regenerate destroyed enamel are an emerging field in regenerative dentistry and are the subject of the work of several research groups [9-11].

Being a static, non-regenerating tissue after eruption, modern oral care focuses mainly on protection and preservation of tooth enamel. For efficient oral care strategies, it is interesting to look at the conditions enamel is exposed to during its lifetime. All calcium phosphates including HAP, Ca_5_(PO_4_)_3_(OH), are soluble in acids, *i.e*. the enamel surface can be attacked by acids and be partially dissolved according to the following equation [1]:

$Ca_{5}(PO_{4}{)}_{3}(\mathrm{OH})+4H^{+}\to 5Ca^{2+}+3\mathrm{HP}O_{4}^{2-}+H_{2}O$

In the oral cavity, enamel is exposed to acids mainly under two conditions: Either enamel can be eroded by acidic food or beverages (acid erosion), or the enamel crystallites can be partially demineralized within the caries process in dental plaque [12].

The critical pH-value for enamel dissolution is about 5.5. However, the solubility of enamel also depends on the amounts of calcium and phosphate ions in saliva or plaque [13]. Chemical analysis of unstimulated saliva reveals that it contains between 0.5 - 2.8 mmol/L calcium and 2 - 22 mmol/L phosphate. Stimulated saliva presents 0.2 - 4.7 mmol/L calcium and 1.5 - 25 mmol/L phosphate [14]. For the HAP constituting of the enamel, it is important that calcium and phosphate are abundant enough to generate a supersaturated state of the saliva. Thus, the enamel surfaces can be remineralized by precipitation of calcium phosphate [14]. A general requirement for remineralization is calcium and phosphate ions passing the protein-rich pellicle layer to reach the enamel surface. The suggested mode of action is that calcium ions interact with glycoproteins (statherin, histatine, acidic proline-rich proteins and others), forming a complex which is termed precipitin [15]. Precipitin is embedded in the pellicle serving as natural calcium-reservoir [15]. However, remineralization from saliva cannot replicate the complex microstructure of enamel. Furthermore, remineralization from saliva is impeded by a longer term acidic milieu [12]. Especially due to modern dietary habits, both acid erosion and caries represent a global disease affecting nearly 100% (dental caries) and 4-100% (dental erosion), respectively [12, 16, 17].

The majority of modern oral care strategies that address these conditions rely on fluorides. Fluoride compounds like Sodium Fluoride (NaF) or Stannous Fluoride (Sn(II)F_2_) are currently the most prominent remineralization systems in oral care. Fluoride ions, F^-^, are believed to enhance the natural enamel remineralization process and to inhibit demineralization [18, 19]. However, the underlying mechanisms of these properties are chemically not yet fully understood which is mainly due to the complex biological processes occurring within the oral cavity, such as interactions of fluorides with proteins and constituents of saliva [20].

Topically applied fluorides are the proven mainstay of contemporary clinical caries prevention and their efficacy has been well documented in numerous clinical studies [21, 22]. Their identified preventive modes of action are manifold and in some aspects still not fully understood. Nevertheless, most experts agree that the single most important caries preventive property of topically applied fluorides is based on accelerating the reintegration of calcium phosphate mostly derived from saliva into demineralized surface lesions at the tooth-bacterial biofilm interface. However, exposure to extraordinary high fluoride levels can cause side effects like fluorosis and bone weakening [12, 23-30]. Therefore, the concentration of fluoride in oral care products is strictly regulated worldwide [22, 23, 31]. For example, in the EU, toothpastes are classified as cosmetic products and a maximum of 1500 ppm fluoride is allowed, and on average toothpastes with fluoride usually sold over the counter contain levels of around 1000 ppm [23, 31]. Besides, caries still develops in high-risk individuals, despite the numerous evidences of fluoride interventions having the most consistent benefit in preventing caries development and decelerating the progression of caries lesions.

While the safety of the proper use of fluoridated toothpastes has been firmly established by numerous studies, dosage and toxicity aspects always have to be considered, particularly in children, where fluoride overdosing may result in the manifestation of mottled enamel or other signs of chronic fluorosis [23].

Fluorides require calcium and phosphate ions from saliva to improve the natural remineralization process. Hyposalivation therefore, a common problem in elderly subjects, may significantly impair the preventive efficacy of topically applied fluorides. For xerostomia patients (*e.g*. induced by medications), oral care products with the addition of calcium and phosphate are recommended in order to compensate a calcium phosphate deficiency [32-35].

To overcome these drawbacks, increasing attention is given to the development of alternative non-fluoride agents that improve remineralization without having any possible side effects on the human body [35, 36]. The clinical use of calcium phosphate in caries protection by contrast is virtually free of comparable dosage and toxicity issues. Ideally, these agents should show equal or superior efficacy in remineralization compared to fluorides and provide an efficient caries protection *in vivo*.

In the last years, biomimetic concepts along these lines have been developed in oral care with the aim to address these issues [1, 9, 37-41]. Especially in the field of enamel remineralization, calcium phosphates have been identified as promising biomimetic alternatives due to their similarity to natural enamel [1, 31, 35, 42-44]. It is assumed that calcium phosphate functions by infiltrating the micropores in early caries lesions, where it acts as crystal nuclei in the remineralization process by continuously attracting large amounts of calcium and phosphate ions from the oral fluids into the lesion, thus promoting natural remineralization processes [31, 42]. Besides remineralizing properties, *in situ* studies with hydroxyapatite have shown anti-adhesive properties that have the potential to be employed for a biomimetic biofilm control [45, 46]. Microorganisms tend to attach to free hydroxyapatite particles originating from toothpaste or mouthrinse and are thus cleared from the oral cavity, since they are deprived from colonizing the enamel [45]. It is also reported that hydroxyapatite forms a protective layer on the enamel surface [46]. Both remineralization concepts, *i.e*. calcium phosphates alone or in combination with fluorides have been realized in modern oral care products [1]. However, it is important to know that combining calcium phosphates with fluorides within a toothpaste formulation may reduce the bioavailability of (ionic) fluoride in the oral cavity. This is due to the reaction of fluoride with calcium forming insoluble compounds such as Calcium Fluoride, CaF_2_, or Fluoroapatite, Ca_5_(PO_4_)_3_F [31, 47, 48].

The in-depth analysis and quantification of enamel remineralization *in vivo* is still a challenge in oral care research because most analytic methods are performed *in vitro*. In general, the remineralization process can be analyzed by different *in vitro* techniques such as Transverse Microradiography (TMR), Scanning Electron Microscopy (SEM) and nano- and microindentation [35, 46, 49-51]. *In situ* studies have gained increasing attention over the last years. In these experiments, individual patients wear bovine or human enamel test specimens, which are carried either on removable oral appliances or fixed on the natural teeth of the human subject. Within the oral cavity these specimens are in contact with saliva and salivary proteins, providing more realistic conditions compared to pure *in vitro* studies. After the exposure within the oral cavity, the specimens can be further analyzed by various analytical methods outside the oral cavity [35, 45, 52]. The calcium phosphate family contains many different compounds such as hydroxyapatite, β-tricalcium phosphate and dicalcium phosphate dihydrate (Table **2**) [2, 53].

There is currently no comprehensive review article available that summarizes all published *in vivo* and *in situ* studies focusing solely on calcium phosphates as biomimetic ingredients without any additional remineralizing agents, such as fluorides. Therefore, the aim of this interdisciplinary review is to bridge this gap by introducing the large spectrum of different calcium phosphates and to summarize the state of the art of knowledge about their effects that have the potential to improve oral care products, such as the biomimetic remineralization of enamel as well as caries prevention with a focus on the latest *in vivo* and *in situ* studies.

## 2 STUDY SELECTION

**Fig. (1):**
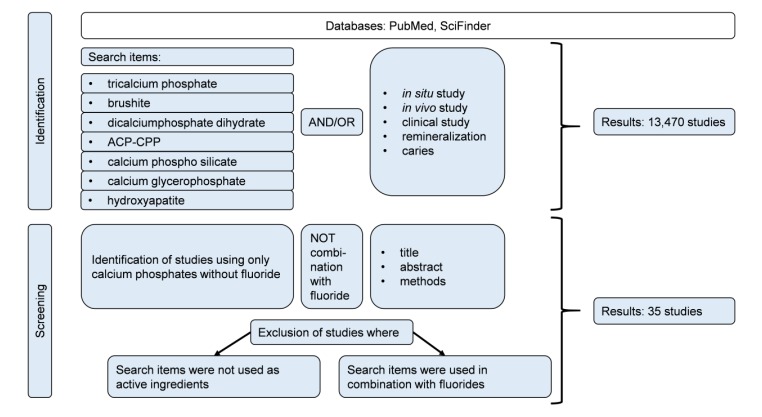
Flow chart of study selection. *In vivo* and *in situ* studies were used for this review. After exclusion of all studies where calcium phosphates were used in combination with fluoride or where calcium phosphates were only mentioned *e.g*. as byproducts within the remineralization process, 35 studies remained and were included in this review.

Books, reviews, and original research papers on *in vivo* and *in situ* studies were included. The databases PubMed^®^ and SciFinder^®^ were used for literature search. Calcium phosphates that are frequently utilized in oral care products were covered in this review and used as search terms alone and together with the following key words: *in vivo*, *in situ*, caries, clinical study, and remineralization. From 13,470 studies found, 35 studies complied with the inclusion criteria and were used for this review (Fig. **1**).

## 3 CALCIUM PHOSPHATES USED IN ORAL CARE

### 3.1 Overview

**Fig. (2):**
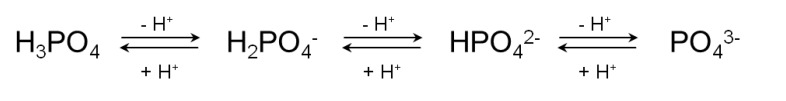
Overview of phosphoric acid and its salts (for the pH-dependence (Table **1**)).

Enamel is organized in a hierarchical structure, starting on the molecular level with the hexagonal hydroxyapatite unit cell. Its properties in terms of dimensions of the crystallographic axes are *a*=9.441 Å and *c*=6.882 Å [54]. Besides hydroxyapatite, many different calcium phosphate phases are known that can be formed under natural and synthetic conditions. This variation in calcium phosphates is brought about by the characteristics of Phosphoric Acid, H_3_PO_4_ [1, 2, 53]. Depending on the pH-value, different stages of deprotonation of H_3_PO_4_ can be observed, *i.e*. H_2_PO_4_^-^ (Dihydrogen Phosphate), HPO_4_^2-^ (Hydrogen Phosphate), and PO_4_^3-^ (Phosphate) (Fig. **2**, Table **1**) [55]. All of these ions can interact with calcium ions and water molecules, leading to many different calcium phosphates (Table **2**).

Thus, for example, the synthesis of stoichiometric hydroxyapatite needs a pH-value >12, otherwise dihydrogen phosphates or hydrogen phosphates will be incorporated into the apatite lattice favoring the formation of a calcium-deficient hydroxyapatite. Synthetic hydroxyapatite is formed by calcium and phosphate ions in a reaction that is comparable to hydroxyapatite formed by ameloblasts during tooth development:

$\mathrm{5C}a^{2+}\mathrm{3P}O_{4}^{3-}+OH^{-}\to Ca_{5}(PO_{4}{)}_{3}(\mathrm{OH})$

Additionally, further ions like Zn^2+^, Sr^2+^, Mg^2+^, Na^+^, CO_3_^2-^ as well as water molecules can be incorporated into the calcium phosphate crystal structure [1, 2, 53]. For example, Roveri *et al.* describe the synthesis and characterization of a biomimetic zinc substituted carbonated HAP mimicking the natural enamel crystallites that can be used for enamel remineralization [40]. In addition to the pH-value and other factors, such as temperature, pressure and additives can affect the formation of the calcium phosphate phases, *e.g*. crystallite morphology and crystallite size [10, 56].

Commonly used calcium phosphates in biomimetic oral care are summarized in Table **3**.

To differentiate the various crystalline calcium phosphate phases, mainly X-ray powder diffraction is utilized. This technique allows identifying a single phase or a mixture of different phases. Rietveld-analysis can additionally be used to calculate the lattice parameters. Amorphous calcium phosphates cannot be routinely analyzed using these techniques due to the absence of a crystalline structure. Further techniques for analysis of chemical composition and microstructure include scanning electron microscopy, energy dispersive X-ray analysis and elemental analysis.

Due to their similarity to natural bones and teeth, synthetic calcium phosphates offer an excellent biocompatibility while being non-toxic. Therefore, besides oral care, they are used in manifold biological and medical applications, for instance the coating of metallic implants, as drug delivery agent or bone cement [2].

### 3.2 Hydroxyapatite (HAP)

**Fig. (3):**
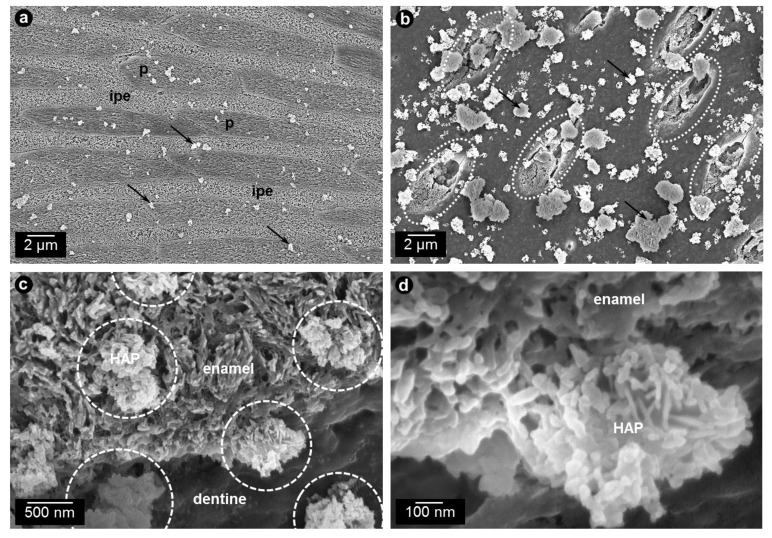
SEM-images of synthetic HAP-particles adhering to bovine enamel (a) and dentin (b) surfaces after having been applied *in vitro*. (**a**) Prismatic (p) and interprismatic enamel (ipe) can be clearly distinguished in this mildly etched sample (0.07 M phosphoric acid, 5 s), the synthetic particles are indicated by arrows. (**b**) Dentin surface of the same sample as (a), both exposed tubuli (encircled with dashed lines) and the bare surface are covered with synthetic HAP-particles (arrows). The affinity of the particles to the surface is higher for dentin than for enamel. (**c**) High magnification micrograph showing the structural similarity of HAP-particles (encircled with dashed lines) to the gently etched naturally formed HAP constituting the enamel. (**d**) Detail images show that the adhesion between enamel and particle is quite firm and individual crystallites at the interface can no longer be clearly associated to one or the other indicating a good mineral-mineral-interaction.

Out of all calcium phosphate phases, HAP, Ca_5_(PO_4_)_3_(OH), has the highest similarity to the natural enamel [1]. Additionally, it has the lowest solubility of all calcium phosphates. HAP can be synthesized in different crystallite morphologies and particle sizes, *i.e*. from nanometer to micrometer size [1]. Commonly used HAP particles in oral care applications are arranged in microclusters [45]. Synthetic HAP particles were shown to interact both with enamel and dentin surfaces (Fig. **3**) where they can unfold their effects such as the reduction of initial bacterial colonization [45, 46, 57].

The interaction of HAP particles with the enamel surface is crucial for the remineralization process. Anti-caries effects were shown in Japanese school children who received either a toothpaste with HAP or a toothpaste without HAP [31, 58]. The results after 3 years show that the mean DMFT-indices were significantly lower in the HAP group (1.45) than in the HAP-free group (3.29) representing a caries-reduction of 56% [31, 58]. In a clinical trial, enamel remineralization rate and enamel resistance against acids were tested using HAP. It could be shown that both improved after three days and three months, respectively [59]. Another clinical study was able to show a reduction of the plaque formation rate of a HAP-containing toothpaste comparable to a amine- and stannous fluoride-containing toothpaste [60]. The investigators also monitored gingival bleeding while probing, which was also reduced by using HAP-toothpaste [60]. In addition to its remineralizing and anti-biofilm characteristics, HAP is used as a biomimetic abrasive in toothpaste formulations [31, 61]. A clinical study was able to show that HAP is as effective as Chlorhexidine (CHX) in plaque reduction and as effective as Fluoride (NaF) in remineralizing initial enamel lesions [62]. The 81 children included in this study were separated equally into three different groups (HAP, CHX, NaF) and used the mouthwashes for at least eight weeks twice a day. After 1, 2, 4 and 6 weeks and two weeks after the end of the study plaque and gingival indices were examined as well as the mineral content of the enamel [62]. The authors conclude that HAP is superior to CHX and NaF mouthrinses in combining the positive effects of two mouthrinses in one application [62]. Two recent studies from Lelli *et al.* and Kensche *et al.* help understanding the action mechanisms of HAP. While Lelli *et al.* were able to detect a protective HAP-layer on enamel surfaces in an *ex-vivo* study [46], Kensche *et al.* used an *in situ* model detecting the anti-adhesive properties of the enamel-like calcium phosphate active ingredient [45]. The formed protective HAP-layer was able to inhibit bacterial colonization on top of the enamel as well as acting as a buffer when acidic attacks occur [45, 46]. The effect is comparable to the effect of chlorhexidine, but the mechanism is not [45]. While chlorhexidine leads to a widespread cell death, HAP simply reduces the microbial load due to anti-adhesive properties [45]. This was also shown before using HAP as active ingredient in a mouthwash [57]. HAP shows the same remineralization properties as fluoride systems (1100 ppm sodium fluoride) *in situ* [35]. Najibfard *et al.* analyzed lesion depth and mineral content of extracted human third molars with and without (control) artificial enamel lesions to test the remineralizing properties of both systems. Each of the 30 participants used each of the toothpastes that were tested (5% HAP, 10% HAP, 1100 ppm NaF) for 28 days followed by a 7-days washout period. The authors conclude by propagating HAP usage instead of fluorides especially for children or patients suffering from xerostomia, because fluorides are potentially toxic and are strictly dependent on the availability of calcium and phosphates [35].

Summarizing the results of these studies, HAP shows equivalent performance compared to the standard use of fluorides in oral care.

### 3.3 β-tricalcium Phosphate (β-TCP)

There are two different tricalcium phosphates, Ca_3_(PO_4_)_2_, known, both of which cannot be found in pure form in nature [2]. While α-tricalcium phosphate can only exist at high temperatures (above 1125 °C), β-TCP is stable at room temperature [2]. β-TCP shows only a moderate solubility in water (25 mg/L at 25 °C) [63]. The size of β-TCP powders differs depending on the milling-procedures, but mostly ranges between 0.01-5 µm [63, 64]. β-TCP is the bioavailable form of tricalcium phosphate that is used in products for medicine and oral care [2, 64].

Remineralization of eroded enamel is performed by calcium and phosphate originating from saliva. By using β-TCP, the concentration of calcium in the saliva can be increased (β-TCP is soluble at pH < 6). An *in vivo* study showed an increase of calcium in the saliva as consequence of acidic attacks and an acidic plaque-pH, when 2.5% β-TCP is used as additional compound in chewing gums [65]. Compared to the control (conventional gum, without an additional calcium source), the pH increased (buffering of acidic attacks) as well as the concentrations of free calcium and free phosphates. Both can be used for remineralization, when the enamel is damaged by acidic attacks. This study also showed a deposition of β-TCP in plaque and saliva becoming available as soon as an acidic attack occurs [65].

When using β-TCP, it is assumed that the concentration/content should be less than 1% (when used in combination with fluoride), because of possible interactions with fluorides, *e.g*. when both agents are used together in toothpastes (see above) [64, 65]. Calcium-phosphate complexes and calcium fluoride can be formed, lowering the bioavailability of both β-TCP and fluoride [64]. However, Functionalized β-tricalcium Phosphate (ƒTCP) technology is currently used in certain brands of toothpaste with 5000 ppm fluoride and fluoride varnish with 5% NaF. In ƒTCP, by milling β-TCP with organic materials (functionalization) the calcium oxides in β-TCP become ‘protected’ by the organic materials (fumaric acid or sodium lauryl sulfate), thus allowing the calcium and phosphate ions of the β-TCP to coexist with fluoride ions in an aqueous dentifrice base (toothpaste or varnish) without premature β-TCP -fluoride interactions [66]. When the toothpaste or varnish is applied intraorally, saliva degrades the protective coating, releasing calcium at the tooth surface, resulting in high fluoride and calcium ions bioavailability on the lesion surface, and subsequent diffusion into the lesion to promote remineralization. The ƒTCP technology has been investigated in mouthrinse and toothpaste tailored for *in situ* remineralization of dental caries [67] and acid erosion [68, 69].

When using β-TCP in toothpastes, surfactants should not contain carboxylic acids or Sodium Lauryl Sulfate (SLS) [64]. Both can coat β-TCP, thus leading to a low extent of bioactive calcium and phosphate. The same can be observed when using polymers or copolymers *i.e*. PVM / MA Copolymer or PEG 8. PVM / MA is a combination of the two polymers (Poly)Methyl Vinyl Ether (PVM) and Maleic Anhydride (MA) and PEG-8 is known as Polyethylenglycol with 8 repetitions of ethylene (–CH_2_–CH_2_–O–) [64]. These limitations lead to the suggestion to use β-TCP as remineralizing agent alone in toothpastes (without fluorides) and to carefully choose surfactants that do not inhibit the bioactivity of calcium. Furthermore, no polymers or copolymers should be used. However, the potential of β-TCP in oral care products for management of oral diseases has not been fully investigated in randomized clinical trials.

### 3.4 Amorphous Calcium Phosphate (ACP)

Amorphous Calcium Phosphates (ACP), Ca*_x_*(PO_4_)*_y_* ∙ *n* H_2_O, are mostly synthesized from the aqueous precipitation of calcium phosphates [2]. The structure of ACP is mostly nano-crystalline, *i.e*. it has a short range order of very small dimensions in the range of a few interatomic distances. X-ray-diffraction confirms an amorphous character. ACP mostly occurs as spherical particles with an average diameter of 20-200 nm that can be visualized by SEM [2]. It is suggested that ACP has apatite-like structures [2]. ACP is well studied and mostly combined with Casein Phosphopeptides (CPP), which stabilize calcium in aqueous solutions [70]. CPP is a natural peptide which can be derived from bovine milk by tryptic digestion of casein containing the protein-sequence Pse-Pse-Pse-Glu-Glu [70, 71]. Furthermore, CPP has been shown to increase the plaque-pH due to enzymatic breakdown of casein and a resulting increase of ammonia [31, 72].

A systematic review from 2014 included 738 studies, eight of which were used for further analysis [73]. These *in vivo* studies intended to analyze the long-term remineralizing effects of CPP-ACP compared to placebo controls [73]. The long-term effects on remineralizing carious lesions were superior compared to placebos and equivalent to fluorides. In addition to these results, CPP-ACP shows no side-effects such as calcified plaque. Using CPP-ACP with fluorides together in one formulation shows conflicting evidence and needs further research [73]. Within a cohort of 191 children, tested CPP-ACP was found to reduce the percentage of *Streptococcus mutans* [74]. While 8% of regular users of a cream containing this calcium phosphate were detected as *S. mutans* carriers, 47% of those who did not use CPP-ACP were colonized with *S. mutans* [74]. These results do not directly show anticariogenic properties of CPP-ACP, but can be interpreted as hint for its ability to reduce potentially cariogenic bacteria. However, clinical relevance of *in vivo* remineralization with significant effects can be corroborated [73, 75]. Another clinical study used CPP-ACP in chewing gums and compared this to a xylitol-containing chewing gum. 60 children (aged 8-12 years) were included in this study and equally distributed within both groups. This study aimed to estimate the salivary flow rate, pH and buffering capacity [76]. While salivary flow rate and pH showed no differences between the two groups, salivary buffer capacity was significantly higher in the ACP-CPP group than in the control group without CPP-ACP [76]. A case report combining ACP-CPP and photo-activated disinfection to treat root caries supports these findings, since root surface caries could be stabilized [77]. Within a clinical trial, CPP-ACP was used to examine the potential of regressing white-spot lesions with orthodontic patients [78]. Even ICDAS II (International Caries Detection and Assessment System) code 2 and 3 lesions were regressed (31%) compared to placebo [78]. Another 10 day *in situ* trial showed ACP-CPP being more effective in remineralizing enamel lesions compared to 1000 ppm fluoride and even 5000 ppm fluoride [79]. Additionally to the demineralization of enamel by acids produced by bacteria, extrinsic acids can also attack the dental hard tissues (erosion). ACP was also confirmed to protect eroded teeth from erosive attacks. The authors describe the use of remineralizing agents in the case of erosion rather as precipitation template formation than as true remineralization [17]. However, ACP (in combination with CPP) is as effective in recovering eroded enamel as fluorides used in typical concentrations within products for cosmetic use [17]. Another study was not able to show reduction of erosion under the given *in situ* conditions by using CPP-ACP [80]. These findings were supported using an *in situ* caries model where the investigators were not able to show a promotion of remineralization through CPP-ACP compared to fluoride control and fluoride/CPP-ACP control [81]. In contrast to that, Kensche *et al.* demonstrated a decrease of acid-induced demineralization, however the effect was inferior to that of fluorides [82]. Contrary to these results, a meta-analysis showed clear short-term remineralizing effects of CPP-ACP as well as long-term reduction of caries [83]. This meta-analysis by Yengopal *et al.* from 2009 used *in situ* and *in vivo* studies concluded the anti-caries efficacy of CPP-ACP [83]. In all cases, the participants used chewing gums supplemented with CPP-ACP. The amount of CPP-ACP ranged between 10.0 mg to 18.8 mg (*in situ*) and 54.0 mg (*in vivo*). Under the chosen *in situ* conditions, the percentage of remineralization with CPP-ACP was about 10% higher compared to no intervention or chewing gum without remineralizing agents, respectively (*p*=0.00001). The *in vivo* study included in this meta-analysis was performed as randomized clinical trial with 2720 participants over 24 months. After this period, the participants who received CPP-ACP supplemented chewing gums showed a reduction of progressing caries of 18% compared to the control group [83].

ACP seems to be a promising candidate for remineralizing initial caries lesions and enamel eroded by acidic attacks, especially if applied directly before, during or after acid-intervention [64, 84]. However, the conditions of ACP synthesis / precipitation and consequently the use in toothpastes, mouth rinses and chewing gums need to be standardized to have the same quality in each application.

### 3.5 Calcium Phosphosilicate (CSPS)

Calcium phosphosilicate (CSPS) is a bioactive glass comprising 45% SiO_2_, 24.5% Na_2_O, 24.5% CaO and 6% P_2_O_5_ [64, 85]. This mineral was originally developed as bone-regenerative material because of its high biocompatibility and the ability to release calcium and phosphate ions [86, 87]. Besides occluding dentinal tubules and consequently desensitizing effects of this calcium phosphosilicate, there are also studies describing remineralizing potential as well as caries prevention and antiplaque-characteristics [64, 85, 88-95]. The active mechanism seems to be the delivery and deposition of calcium- and phosphate-ions that form a crystalline carbonated-apatite layer [31, 85].

Tai *et al*. Investigated the Plaque Index (PLI) and Bleeding Index (BLI) within a study with 100 subjects in a RCT over a six-week time period [89]. In this study, CSPS was tested against a placebo-control and showed significant improve of oral health measured by a reduction of PLI and GBI [89]. Nevertheless, the mode of action was not clarified by this study.

Additionally, two *in situ* studies both from Parkinson *et al.* from 2017 used sodium monofluorophosphate with different concentrations as positive control and 5% CSPS as test dentifrice. They used also different concentrations of sodium monofluorophosphate in both studies (ranged from 0% to 0.15%) [94, 95]. The authors conclude in both studies no detectable improvements of 5% CSPS (alone or in comparison with sodium monofluorphosphate) compared to the positive (0.15 ppm sodium monofluorphosphate, 0% CSPS) or the negative control (0 ppm sodium monofluorphosphate, 0% CSPS) [94, 95].

CSPS as active compound in toothpastes seems to act as a calcium-reservoir that can be used for remineralization of demineralized enamel or dentine. Clinical studies with CSPS alone (not in combination with any fluorides) are needed to test the outcome for caries prevention *in vivo*. Plaque reduction and clear improvement of oral (gingival) health can be observed, when CSPS is used. *In-situ* studies were able to show non-inferiority to fluorides in case of remineralization.

Unlike the bioglass described above, which required the addition of fluoride compounds such as sodium monofluorophosphate into the toothpaste formulation, a fluoride-containing bioactive glass was recently introduced as a caries remineralizing and preventive additive in toothpastes. Fluoridated Bioglass (f-BG) has fluoride, strontium, potassium and zinc incorporated within the glass itself, thus enabling simultaneous delivery of Sr^2+^, Ca^2+^, PO_4_^3−^ and F^−^ ions into the initial caries lesions to promote remineralization by formation of a partially fluoridated crystal structure, zinc ions for bactericidal function, and potassium as a desensitizing agent. Having the fluoride incorporated within the glass prevents the risk of premature reaction of fluoride and calcium ions to Calcium Fluoride (CaF_2_), which reduces the bioavailability of the two ions [96, 97]. However, the lack of clinical studies does not permit any firm conclusions on their effectiveness.

### 3.6 Calcium Glycerophosphate (CGP)

Calcium Glycerophosphate, C_3_H_7_CaO_6_P, (CGP) is the salt of glycerophosphoric acid. It is typically used as a food ingredient and a nutrition supplement [98]. The first studies from 1972 evaluating the cariostatic effect of this organic calcium phosphate by Bowen used CGP as a nutrient supplement [99]. Here, a group of monkeys (*n*=5) received 1% CGP as nutrient supplement. The control group (*n*=6) was fed with the same carbohydrate-rich diet as the experimental group, but without CGP. After a period of 30 months, the experimental group showed 8 carious lesions compared to 47 of the control group [99]. Despite higher Ca^2+^-levels in the plaque of the experimental animals as well as higher pH-values after 20 min of sucrose administration, the total content of proteins and phosphate was the same in both groups [99]. Brook *et al.* used CGP as nutrient supplement, too [100]. Within a cohort of 14 children, the mean plaque levels were estimated between a group of children receiving 1% CGP 4 times daily and a group without any specific treatment. While plaque levels were increased in the experimental group, Ca^2+^ was not different [100]. An even smaller study sample (n=8) did not clean their teeth for 18 days and rinsed with a 50%-sucrose solution [101]. Optical changes were characterized as demineralization and could not be reduced by the addition of CGP (1%). Even topical applications of sodium fluoride (2%) were not able to inhibit these changes [101]. Another study determined the accumulation of CGP in the dental plaque by having three different mouthrinse-interventions: (1) No CGP, (2) 0.5% CGP and (3) 1.5% CGP [102]. The concentration of phosphate was significantly greater in the plaque in (3) compared to (1), indicating a higher potential for buffering acidic attacks [102].

Forward [103] and Lynch [104] both postulated non-fluoride agents to modify the dental plaque with CGP or other agents [103] and enhance remineralization by enhancing calcium levels in the dental plaque to be promising factors for reducing dental caries [103, 104].

## CONCLUSION

Calcium phosphates represent a group of common agents used in oral care that can be considered biomimetic due to the fact that the mineral phase of teeth consists of the same basic compound. Fluorides have been proven to efficiently reduce caries, but due to persistent high prevalence of this disease and the possible side effects of fluorides under certain conditions there is still a need to improve and also explore alternative ways for the prevention of tooth decay [36]. Several different calcium phosphates are already clinically proven as anti-caries agents. Therefore, both fundamental studies on interaction mechanisms and further clinical studies on alternative, biomimetic agents are needed to help reduce the still high global caries prevalence. Among the different calcium phosphates, HAP and ACP have been most intensively studied and their efficiency in oral care has been shown in various *in situ* and *in vivo* studies. Therefore, they are clear favorites for the development of innovative biomimetic oral care concepts. However, the potential of other calcium phosphates for oral care applications should be analyzed in further studies. HAP and ACP show manifold activity in reduction of biofilms and remineralization of enamel and dentin. In contrast to fluorides, calcium phosphates are ideally suited especially for young children and individuals suffering from hyposalivation.

In conclusion, calcium phosphates represent a promising innovative approach for daily oral care that will broaden the range of future treatments in preventive dentistry.

## LIST OF ABBREVIATIONS

**Table:** 

**Abbreviation**	**Name**
ACP	Amorphous Calcium Phosphate
α-TCP	α-Tricalcium Phosphate
β-TCP	β-Tricalcium Phosphate
BLI	Bleeding Index
CDHA	Calcium-Deficient Hydroxyapatite
CHX	Chlorhexidine
CPP	Casein Phosphopeptides
DCPA	Dicalcium Phosphate Anhydrate / Monetite
DCPD	Dicalcium Phosphate Dihydrate / Brushite
DMFT	Decayed Missing Filled Teeth
ƒTCP	Functionalized β-Tricalcium Phosphate
HAP	Hydroxyapatite
MCPM	Monocalcium Phosphate Monohydrate
MCPA	Monocalcium Phosphate Anhydrate
OCP	Octacalcium Phosphate
PLI	Plaque Index
RCT	Randomized Clinical Trial
SCPP	Secretory Calcium Binding Phosphoprotein
SEM	Scanning Electron Microscopy
TEM	Transmission Electron Microscopy
TTCP	Tetracalcium Phosphate
TMR	Transverse Microradiography

## Figures and Tables

**Table 1:** pH-dependency of phosphoric acids and its salts [55]. All these ions can interact with calcium ions, which lead to a huge variety of calcium phosphate phases.

**pH**	**Mol-%**
-0.5	100% H_3_PO_4_
2	50% H_3_PO_4_, 50% H_2_PO_4_^-^
4.5	100% H_2_PO_4_^-^
6	90% H_2_PO_4_^-^, 10% HPO_4_^2-^
8	10% H_2_PO_4_^-^, 90% HPO_4_^2-^
9.5	100% HPO_4_^2-^
12	50% HPO_4_^2-^, 50% PO_4_^3-^
14.5	100% PO_4_^3-^

**Table 2:** Overview of different calcium phosphates with biological importance [2]. Calcium phosphates frequently used in oral care products are highlighted (*).

**Name of the Calcium Phosphate Phases Including Abbreviations**	**Chemical Formula**
Monocalcium phosphate monohydrate (MCPM)	Ca(H_2_PO_4_)_2_ ∙ H_2_O
Monocalcium phosphate anhydrate (MCPA)	Ca(H_2_PO_4_)_2_
Dicalcium phosphate dihydrate (DCPD, “brushite”)	CaHPO_4_ ∙ 2 H_2_O
Dicalcium phosphate anhydrate (DCPA, “monetite”)	CaHPO_4_
Octacalcium phosphate (OCP)	Ca_8_(HPO_4_)_2_(PO_4_)_4_ ∙ 5 H_2_O
α-tricalcium phosphate (α-TCP)	α-Ca_3_(PO_4_)_2_
β-tricalcium phosphate (β-TCP) *	β-Ca_3_(PO_4_)_2_
Amorphous calcium phosphate (ACP) *	Ca*_x_*(PO_4_)*_y_* ∙ *n* H_2_O
Calcium-deficient hydroxyapatite (CDHA)	Ca_10-_*_x_*(HPO_4_)*_x_*(PO_4_)_6-_*_x_*(OH)_2-x_ (0<*x*<1)
Hydroxyapatite (HAP) *	Ca_5_(PO_4_)_3_(OH)
Tetracalcium phosphate (TTCP)	Ca_4_(PO_4_)_2_O

**Table 3:** Overview of *in vivo* and *in situ* studies that were identified for the chosen calcium phosphates including aims, methods, and main outcomes. Please note that the presented calcium phosphates are mostly incorporated into oral care products like toothpastes and mouthrinses.

**Name of the Calcium Phosphate Phases Including Abbreviations**	***In vivo* Studies**	***In situ* Studies**
Hydroxyapatite (HAP), Ca_5_(PO_4_)_3_(OH)	**Kani *et al.* [****58****]** *Aim:* Analysis of the anti-caries effects of a hydroxyapatite toothpaste compared to a placebo toothpaste *Methods:* DMFT-indices *Outcome:* HAP reduces caries within a cohort of Japanese schoolchildren	**Kensche et al. [****45****]** *Aim:* Analysis of hydroxyapatite particles in oral biofilm management *Methods:* DAPI and live/dead staining, SEM *Outcome:* HAP particles reduce initial biofilm formation on enamel surfaces comparable to chlorhexidine
	**Makeeva *et al.* [****59****]** *Aim:* Analysis of caries resistance of tooth enamel and teeth sensitivity after using a hydroxyapatite-toothpaste *Methods:* Assessment of enamel remineralization rate, dynamics of enamel acid resistance and teeth sensitivity Outcome: HAP remineralized enamel and led to an increased acid resistance	**Hannig *et al.* [****57****]** *Aim:* Analysis of a hydroxyapatite mouthwash in oral biofilm management *Methods:* DAPI and live/dead staining *Outcome:* HAP-containing mouthrinse reduces initial biofilm formation comparable to chlorhexidine
	**Harks *et al.* [****60****]** *Aim:* Analysis of a hydroxyapatite-toothpaste in periodontitis patients *Methods:* Plaque formation rate, plaque control record, gingival index, bleeding on probing, pocket probing depth *Outcome:* HAP toothpaste improves periodontal health	**Najibfard *et al.* [****35****]** *Aim:* Analysis of the potential of a hydroxyapatite toothpaste in the remineralization of early caries lesions *Methods:* Microradiography *Outcome:* HAP remineralizes initial caries lesions comparable to fluoride
	**Lelli *et al.*** [46]: *Aim:* Analysis of the potential of a hydroxyapatite toothpaste in remineralization of enamel *Methods:* SEM, TEM, EDX, XRD *Outcome:* HAP builds a protective layer on the enamel surface	
	**Hegazy *et al.* [****62****]** *Aim:* Analysis of a hydroxyapatite mouthwash in controlling plaque accumulation, gingivitis and remineralization *Methods:* Plaque and gingival indices, DIAGNOdent *Outcome:* HAP reduces plaque and gingival index and remineralizes early caries lesions	
β-tricalcium phosphate (β-TCP), β-Ca_3_(PO_4_)_2_	**Vogel *et al.* [****65****]** *Aim:* Analysis of calcium phosphate concentrations in plaque, plaque fluid and saliva *Methods:* Analysis of pH, free and total calcium, total phosphate Outcome: Deposition of calcium in the plaque and saliva after using β-TCP chewing gum	None
Amorphous calcium phosphate (ACP), Ca*_x_*(PO_4_)*_y_* ∙ *n* H_2_O	**Li *et al.* [****73****]** This review shows that CPP-ACP has a significant remineralizing effect	**Lussi & Ganss [****17****]** In this overview, the authors state CPP-ACP to be as effective as low-concentrated fluorides (cosmetic use) and less effective than high concentrated fluorides in preventing and remineralizing eroded lesions
	**Pukallus *et al.* [****74****]** *Aim:* Analysis of a CPP-ACP cream in reducing mutans streptococci colonization and prevent early childhood caries *Methods:* Reduction in mutans streptococci colonization *Outcome:* CPP-ACP reduces mutans streptococci with 24 month old children, but not the caries prevalence	**Wiegand & Attin [****80****]** *Aim:* Analysis of the effect of milk and CPP-ACP pastes on erosion *Methods:* Profilometry *Outcome:* CPP-ACP is not effective in reducing enamel and dentine loss
	**Hedge *et al.* [****76****]** *Aim:* Comparison of salivary flow rate, pH and buffering capacity before/after chewing a CPP-ACP gum *Methods:* Collection of unstimulated and stimulated saliva; analysis of salivary flow rate, pH, and buffering capacity *Outcome:* CPP-ACP used in chewing gums increases salivary buffer capacity compared to a chewing gum without	**Meyer-Lückel *et al.* [****81****]** *Aim:* Evaluate the remineralizing potential of a fluoride-free CPP-ACP-containing cream after the use of a fluoride-toothpaste compared to the prolonged use of a fluoride-toothpaste *Methods:* Transversal microradiography *Outcome:* CPP-ACP is less effective in remineralizing caries lesions compared to prolonged application of fluoride toothpaste
	**Vlacic *et al.* [****77****]** *Aim:* Management and treatment of root caries *Methods:* Laser fluorescence *Outcome:* This case report shows CPP-ACP to be effective in stabilizing root caries lesions	**Kensche *et al.* [****82****]** *Aim:* Influence of calcium phosphate based products on erosion *Methods:* Quantitative analysis of calcium and phosphate, SEM, TEM *Outcome:* Improvement of erosion protective properties using CPP-ACP was not as high as with fluorides
		**Yengopal *et al.* [****83****]** This systematic review shows short-term remineralizing effects and caries preventing long-term effects
	**Bailey *et al.* [****78****]** *Aim:* Effects of a remineralizing cream in post-orthodontic subjects *Methods:* ICDAS II *Outcome:* Regression of white spot lesions (ICDAS II code 2 and 3) compared to placebo (31%)	**Shen *et al.* [****79****]** *Aim:* Analysis of the potential of calcium phosphate based products to remineralize enamel lesions *Methods:* Quantitative analysis of calcium, phosphate and fluoride; transverse microradiography *Outcome:* Enamel lesion remineralization of CPP-ACP was significantly higher compared to placebo, 1000 ppm fluoride and 5000 ppm fluoride; highest remineralization was identified with a combination of CPP-ACP and 900 ppm fluoride
Calcium phosphosilicate (CSPS), 45% SiO_2_, 24.5% CaO, 24.5% Na_2_O, 6% P_2_O_5_	**Tai *et al.* [****89****]** *Aim:* Analysis of anti-gingivitis and anti-plaque effects of a bioactive glass-containing toothpaste *Methods:* Plaque index, gingival bleeding index *Outcome:* Bioactive glass-containing toothpaste significantly reduces gingival bleeding and supragingival plaque compared to a placebo	**Parkinson *et al.* [****94****]** *Aim:* Investigate the effect of CSPS alone and in addition with SMFP on the enamel remineralization Methods: Surface microhardness (Knoop hardness) *Outcome:* CSPS and SMFP have both the same cariostatic effect
		**Parkinson *et al.* [****95****]** *Aim:* Elucidate potential interactions of CSPS on the efficacy of SMFP to promote remineralization *Methods:* Surface microhardness and transverse microradiography *Outcome:* CSPS has the same remineralizing effect as fluoride (SMFP) and does not improve the cariostatic effect of SMFP
Calcium glycerophosphate (CGP), C_3_H_7_CaO_6_P	**Bowen *et al.* [****99****]** *Aim:* Test the caries activity after addition of CGP to the diet of monkeys *Methods:* 11 monkeys received a carbohydrate-rich-diet. 5 monkeys were additionally fed with 1% CGP within the diet. After 30 months, carious lesions were identified *Outcome:* CGP as monkey diet addition shows a significantly cariostatic effect compared to no intervention	None
	**Brook *et al.* [****100****]** *Aim:* Determine the calcium and phosphate concentration in dental plaque after consuming milk cereal tablets with 1% CGP *Methods:* 14 children consumed 4 times a day for 3 months tablets with 1% CGP. Before, in between and after the study plaque samples were analyzed *Outcome:* No increase in calcium-levels in the plaque	
	**Edgar *et al.* [****105****]** *Aim:* Identify the anti-caries effect of CGP *Methods:* 8 volunteers rinsed for 18 days with 50% sucrose solution (9 times daily for 2 min.). Half of the time (9 days) CGP (1%) was added to the solution. After this study period, NaF (2%) was topically applied *Outcome:* Neither CGP nor 2% fluoride application were able to inhibit demineralization of teeth that were not cleaned for 18 days and exposed to sucrose	
	**Wycoff *et al.* [****102****]** *Aim:* Effect of mouthrinses with CGP on the amount and chemical composition of dental plaque *Methods:* 60 children between 13 and 16 years of age were separated into three groups: 10 mL of mouthrinse twice daily with (i) 0,5% CGP, (ii) 1,5% CGP and (iii) placebo. Duration: 8 weeks. Analysis of plaque weight and chemical composition *Outcome:* CGP as mouthrinse shows increased phosphate in the plaque	
